# Dendritic Cell-Restricted Progenitors Contribute to Obesity-Associated Airway Inflammation via Adam17-p38 MAPK-Dependent Pathway

**DOI:** 10.3389/fimmu.2020.00363

**Published:** 2020-02-28

**Authors:** Anil Kumar Jaiswal, Sangeet Makhija, Natalie Stahr, Maninder Sandey, Amol Suryawanshi, Ankit Saxena, Pradeep K. Dagur, J. Philip McCoy, Stewart J. Levine, Amarjit Mishra

**Affiliations:** ^1^Laboratory of Lung Inflammation, College of Veterinary Medicine, Auburn University, Auburn, AL, United States; ^2^Department of Pathobiology, College of Veterinary Medicine, Auburn University, Auburn, AL, United States; ^3^Flow Cytometry Core Facility, Division of Intramural Research, National Heart, Lung, and Blood Institute (NHLBI), National Institutes of Health (NIH), Bethesda, MD, United States; ^4^Laboratory of Asthma and Lung Inflammation, Division of Intramural Research, National Heart, Lung, and Blood Institute (NHLBI), National Institutes of Health (NIH), Bethesda, MD, United States

**Keywords:** dendritic cell-restricted progenitors, CDPs, obesity, asthma, Adam17, p38 MAPK pathway

## Abstract

Proliferation of dendritic cell (DC)—restricted progenitor cells in bone marrow compartment is tightly regulated at steady state and responds to multiple tissue-specific triggers during disturbed homeostasis such as obesity. DCs in the lung stem from a rapidly dividing DC-restricted progenitor cells and are effective at generating adaptive immune responses in allergic airway inflammation. Precisely, how DC-restricted progenitor expansion and differentiation are influenced by airway inflammation to maintain constant supply of myeloid DCs is poorly understood. Here we show that a high fat diet (HFD) induces oxidative stress and accelerates the expansion of DC- restricted progenitor cells in bone marrow and correlates with persistent induction of p38 mitogen activated protein kinase (MAPK), which is blocked with a selective p38α/β MAPK inhibitor. Mice fed a HFD and sensitized to inhaled allergen house dust mite (HDM) led to alterations of DC- restricted progenitor cells that were characterized by increased expansion and seeding of lung DCs in airway inflammation. Mechanistically, we establish that the expansion induced by HFD dysregulates the expression of a disintegrin and metallopeptidase domain 17 (Adam17) and is required for p38 MAPK activation in DC-restricted progenitors. These results demonstrates that obesity produces persistent changes in DC precursors and that elevation of Adam17 expression is tightly coupled to p38 MAPK and is a key driver of proliferation. Altogether, these data provide phenotypic and mechanistic insight into dendritic cell supply chain in obesity-associated airway inflammation.

## Introduction

The prevalence of obesity continues to rise at a staggering rate in developed countries and is considered to be a proven risk-factor for both allergic and non-allergic asthma (1–3). Asthma severity appears to be increased in the obese and alters the response to controller therapy leading to greater healthcare utilization and reduced quality of life. In clinic, asthma in obese are distinguished by early-onset allergic and late-onset non-allergic phenotypes (4, 5) manifested by elevated IgE and serum eosinophil levels. Dietary high fat is one of the key factor to the development of obesity in humans. Previous animal studies have demonstrated various mechanism for unraveling the intricacies of high fat diet (HFD)-induced or HFD-exacerbated asthma (6, 7). A HFD qualitatively or quantitatively modifies lung adaptive immune response and airway inflammation by sensitization to inhaled allergen (8, 9). Among various aberrant systemic changes, HFD promotes low-grade inflammation initiated via multiple tissue-specific triggers (10, 11), circulating inflammatory mediators, and gut microbiota (12, 13). HFD alters bone marrow (BM) environment and homeostasis thereby influence leukocytosis, a hallmark of obesity-associated diseases (14–17). As such, HFD affects both the numbers and physiological function of lung immune cells stem from myeloid and lymphoid precursors in the BM (18, 19). As a consequence, to meet the enhanced demand of these cells the BM-lung axis are particularly salient in controlling allergic airway inflammation (20).

Airway dendritic cells (DCs) are professional antigen presenting cells and maintains lung immunity in response to inhaled allergens. The average resident-time of airway DCs in the lung are short, suggesting that these cells are continuously replenished by the hematopoietic system (21–24). Myeloid-derived lung DCs consist at least of three main populations: conventional or classical DCs (cDCs) that specifically express the transcription factor Zbtb46 (25, 26); type-I interferon-producing plasmacytoid DCs (pDCs) (27); and monocyte-derived DCs (moDCs) (28). Lung cDCs are further categorized as CD103^+^ cDCs and CD11b^+^ cDCs subpopulation, both instigate allergic airway inflammation (29, 30). Recent research studying the development pathways of DCs specifically focused on cDCs and pDCs that stem from clonogenic common DC progenitors (CDPs) in the BM (31, 32), in contrast to macrophage and moDCs that derived from macrophage-DC progenitors (MDPs) or precursor intermediate granulocyte macrophage progenitors (GMPs) (33). Research identified a direct impact of diet-induced alteration of hematopoietic stem cells (HSCs) and progenitors in BM compartment (34–36). Although obesity has also been associated with self-renewal, exhaustion, and mobilization of HSCs, its impact on DC-restricted progenitors in BM compartment and output to promote airway inflammation remains poorly understood. In this study we show that HFD-induced obesity alters the BM environment and DC-restricted progenitor proliferation and functional properties. We show that HFD promotes CDP proliferation in response to oxidative stress via activation of p38 MAPK signaling pathway and further accelerates lung DCs accumulation in response to sensitization of inhaled allergen. Molecularly, we establish that a disintegrin and metallopeptidase domain 17 (Adam17) is a key regulator of DC-restricted progenitors expansion and p38 activation in obesity. Altogether, this work identifies a key molecular mechanism by which obesity shapes the DC-restricted precursors and expansion in BM, which instigate DC-mediated allergic airway inflammation.

## Materials and Methods

### Reagents

High fat (TD.06414; 60 kcal% fat) or chow diet (TD.94048; 10 kcal% fat) Teklad custom diets were purchased from Envigo (Denver, Colorado, USA). HDM (*Dermatophagoides pteronyssinus*) extract was purchased from Greer Laboratories, Lenoir, NC, USA as a freeze-dried preparation (Item no. B82). Quantitative ELISA kits for measurements of CCL24, IL5, IL13, and IFN-γ were from R& D Systems (Minneapolis, MN, USA). Click-iT EdU (5-ethynyl-2′- deoxyuridine)—Alexa Fluor 647 kit for measurement of cell proliferation were from Life Technologies Corporation (Grand Island, NY, USA). The p38 MAPK selective inhibitor Losmapimod (GW856553X) was from Selleckchem (Houston, TX, USA). Adam17 inhibitor TMI 1 was from Tocris (Minneapolis, MN, USA). The intracellular signaling membrane array kit was from Cell Signaling Technology (Danvers, MA, USA).

#### Diet-Induced Obese Mice and Allergen Sensitization and Challenge

Sixteen weeks old HFD (DIO, stock# 380050) and CF control (stock# 380056) males (all in C57BL/6J background) were purchased from Jackson Laboratories (Bar Harbor, MA, USA) and were maintained in-house with 60 kcal% fat (TD.06414) or 10 kcal% fat (TD.94048; Envigo, Inc.) for entire duration of allergen sensitization and challenge protocol. Diets were replaced periodically with the fresh diet for an every 5 days interval. In separate experiments, mice were sensitized by intraperitoneal injection of HDM (100 μg) emulsified in 200 μl of PBS containing 3 mg of aluminum hydroxide (Sigma-Aldrich) on days 0 and 4. Mice were challenged by intranasal administration of HDM (100 μg) in a volume of 40 μl on days 8, 10, 12, and 14 and end points were analyzed on day 15. All animal experiments were approved by the Auburn University Animal Care and Use Committee.

#### Flow Cytometry

Cell surface staining and CDP isolation were performed as described earlier (31). In brief, BM cells were collected by crushing leg bones, treating with ACK lysis buffer to lyse red blood cells, and purifying the cells. Enrichment of cells were done according to the Easy Sep mouse hematopoietic progenitor cell isolation kit (Stem Cell Technology). Cells were then stained and CDPs were analyzed or flow sorted using FITC-conjugated-Lineage cocktail, FITC-conjugated Sca1 (*clone* D7), BV650-conjugated c-Kit (*clone* ACK-2), APC-conjugated CD115 (*clone* AFS98), PE-conjugated Flt3 (*clone* A2F10), PE/Cy5- conjugated CD34 (*clone* MEC.14.7), and PE/Cy7- conjugated CD16/32 (*clone* 93) antibodies.

Lung myeloid cells were identified using antibodies against rat anti-mouse CD45 efluor 450 (*clone* 30-F11), CD11c-APC-Cy7 (*clone* N418), MHCII-PE-Cy7 (*clone* M5/114), SiglecF-Alexa Fluor 647 (*clone* E50-2440), CD103-PerCP-Cy5.5 (*clone* M290), CD11b-e-Fluor 660 (*clone* M1/70), CD64-PE (*clone* X54-5/7.1), CD24- Alexa Fluor 700 (*clone* M1/69), PDCA1-Alexa Fluor 488 (*clone* e-Bio 927), all from eBiosciences. Tregs were analyzed using CD3-Alexa Fluor 647 (*clone* 17-A2), CD4-FITC (*clone* GK1.5), and CD25-PE-Cy7 (*clone* PC61.5) from eBiosciences. For quantification of intracellular Foxp3, cells were fixed and permeabilized with Foxp3 staining buffer and reacted with a Foxp3-PE antibody (*clone* NRRF-30). Cellular debris was excluded using forward light scatter/side scatter plot. Data were acquired on a LSR-II (BD Biosciences) equipped with 407, 488, 532, and 633 laser lines. Results were analyzed with the Flow Jo software version 10 (Treestar, San Carlos, CA, USA), using FMO (fluorescence minus one) as controls.

#### Array Hybridization

CDPs from HFD and CF mice were extracted for proteins and prepared (PathScan intracellular signaling membrane kit, Cell signaling). Cell lysate were incubated with array membrane and reacted to detection antibody cocktail to develop chemiluminescent signal.

#### *In vivo* Assays

For assessment of cell cycle profile of CDPs in BM, we use the Invitrogen ClickiT EdU-AF 647 kit and DAPI (10 μg ml^−1^, Invitrogen) DNA staining dye. Mice were injected i.p. with 50 μg of EdU in sterile PBS and BM cells were collected 18 h after injection. Cells were stained with the following surface markers: FITC-conjugated-Lineage cocktail, FITC-conjugated Sca1 (*clone* D7), BV650-conjugated c-Kit (*clone* ACK-2), APC-conjugated CD115 (*clone* AFS98), and PE-conjugated Flt3 (*clone* A2F10) and fixed with the clickiT fixatives for 15 min. Cells were washed twice and permeabilized with saponin. Cells were washed and incubated with DAPI solution for 2 hat 4°C before each FACS analysis.

#### *Ex vivo* Measurements of Reactive Oxygen Species

Freshly isolated BM cells were enriched for Lin^−^, Sca-1^+^ c-kit^+^ (LSK^+^) cells by immunomagnetic selection using EasySep mouse hematopoietic progenitor cell isolation kit (Stemcell Technology) and stained with APC-conjugated CD115 (*clone* AFS98), PE-conjugated Flt3 (*clone* A2F10) antibodies. Cells were incubated with 5 μM of the redox-sensitive probe CM-H_2_DCFDA, [5- (and−6) chloromethyl-2′ 7′-dichlorohydrofluorescein diacetate, acetyl ester; Molecular probes, Life Technologies] for 30 min at 37°C. The stable fluorescent adduct that was produced by oxidation of CM-H_2_DCFDA in presence of intracellular reactive oxygen species in LSK^+^ CD115^+^ Flt3^+^ cells was quantified by an increase in fluorescence in the fluorescein channel by flow cytometry.

#### *In vitro* Assays

To study the effect of p38 MAPK activation and Adam 17 signaling pathway, BM progenitor cells and splenic DCs were isolated and enriched with the Easy Sep mouse hematopoietic progenitor cell isolation kit and pan DC kit, respectively (Stem Cell Technology). 200,000 cells were cultured in 96-well plates overnight in Iscove's modified Dulbecco's medium supplemented with 10% (vol/vol) FCS, 2- mercaptoethanol (50 μm), sodium pyruvate (1 mM), penicillin (100 U ml^−1^ l) streptomycin (100 μg ml^−1^), mouse GM-CSF (20 ng ml^−1^) and human Flt3L-Ig (100 ng ml^−1^). Cells were incubated in presence or absence of the selective inhibitor losmapimod (20 μM, GW856553X, Selleckchem) or Adam17 inhibitor (50 μM, Tocris). Cultures were stimulated with 5 μg ml^−1^ of phorbol 12-myristate 13-acetate and ionomycin (PMA) and 5 μg ml^−1^ ionomycin (Sigma-Aldrich, St. Louis, MO, USA) for 15 min before fixation and permeabilization with BD Phosflow Perm Buffer III (BD Biosciences) or for EdU staining. Cells were stained with mouse antibodies against CD11c-APC-Cy7 (N418), CD11b-BV650 (M1/70), PDCA1-FITC (1A8), MHCII-PE-Cy7 (M/114) from BioLegends and p38 MAPK-Alexa Fluor-647 (pT180/pY182) (BD Biosciences) and acquired by flow cytometry. p38 MAPK phosphorylation were assessed on gated population of LSK^+^CD115^+^ Flt3^+^ (CDPs) cells or CD11c^+^ CD11b^+^ DCs.

#### Analysis of Airway Inflammation

Bronchoalveolar lavage (BAL) cell counts were performed and differential cell counts were enumerated on Wright- Giemsa-stained cytospin slides. BAL levels of CCL24 and lung IL5, IL13, and IFN-γ cytokines were determined using quantitative ELISA kits from R& D Systems (Minneapolis, MN, USA). Lung sagittal sections were cut to thickness of 5 μm and stained with hematoxylin and eosin or periodic acid Schiff (PAS) for histology. HDM-specific IgE and IgG1 levels in plasma were determined using biotinylated anti-mouse IgE or anti-mouse IgG1 (Pharmingen, San Jose, CA, USA) at a concentration of 2 μg ml^−1^ for 1 h and the amount of bound HDM-specific antibody was determined using TMB substrate.

#### qRT-PCR

RNA was isolated from cultured progenitor cells or splenic DCs using trizol reagent (Life Technologies, Grand Island, NY, USA) and cDNA was generated using a High Capacity RNA- to-cDNA kit (Applied Biosystems). The cDNA was pre-amplified using previously described primers: Zbtb46: *forward-5*′*-AGAGAGCACATGAAGCGACA-3*′*, reverse-5*′*-CTGGCTGCAGACATGAACAC-3*′, Batf3: *forward-5*′*-CAGACCCAGAAGGCTGACAAG-3*′*, reverse- 5*′*-CTGCGCAGCACAGAGTTCTC-3*′. Flow sorted CDPs were extracted for RNA using Arcturus PicoPure RNA isolation kit (Applied Biosystems) and cDNA was prepared. The differential expression of cell cycle phase genes and Adam17 were quantified using Taqman qRT–PCR primers (Thermo Fisher Scientific) (Table 1). After amplification, Cq values were obtained and analyzed using DataAssist software (Applied Biosystems).

**Table 1:** List of primers used in this study.

	**Assay ID**	**Gene symbol**	**Gene name**
1.	Mm00438070_m1	Ccnd2	Cyclin D2
2.	Mm01266311_m1	Ccne1	Cyclin E1
3.	Mm00438084_m1	Ccng1	Cyclin G1
4.	Mm01171453_m1	Ccnb2	Cyclin B2
5.	Mm00432385_m1	Ccnf	Cyclin F
6.	Mm04207341_m1	Cdkn1a	Cyclin-dependent kinase inhibitor 1A (P21)
7.	Mm02619580_g1	Actb	Actin beta
8.	Mm00456428_m1	Adam 17	A disintegrin and metallopeptidase domain 17

#### RNA Sequencing

Fifty thousand CDPs were isolated from HFD and CF mice by FACS sorting directly into lysis buffer of Arcturus PicoPure RNA isolation kit for total RNA extraction and purification. Four independent replicates were isolated in parallel to ensure reproducibility and statistical analysis. Quality of RNA was assessed before being processed for library preparation using Bioanalyzer (Agilent Technologies). The whole transcriptome was amplified and library was constructed by using NuGen Ovation Solo RNA-Seq System (Mouse part no. 0501) that integrates NuGEN's Insert-Dependent Adaptor Cleavage (InDA-C) technology to provide targeted depletion of unwanted transcripts. This resulted in a significant reduction in sequencing reads derived from rRNA and any other targeted transcripts for more efficient use of sequencing resources. Quantitative assessment of library was done using Qubit 2.0 fluorometer (Invitrogen) and evaluated on the high-sensitivity DNA chip (Agilent Technologies). Libraries were sequenced on a HiSeq 3000 platform (Illumina) using the pair-end 75 bp sequencing strategy.

#### Bioinformatics Analyses

RNA-Seq data were aligned with the reference genome using the latest version of HISAT2, which sequentially aligns reads to the known transcriptome and genome using the splice-aware aligner built upon HISAT2 (37). A rigorous validation demonstrated this procedure outperforms other splice-aware aligners for accurately mapping simulated spliced reads, with only a slightly lower alignment reads. Uniquely mapped paired-end reads were then used for subsequent analyses. String Tie was used for transcript assembly and abundance estimation in the non-novel mode using the latest version of GENCODE comprehensive gene annotations. Raw read counts and FPKM (Fragments Per Kilobase of transcript per Million) mapped reads abundance were estimated at the transcript-level as well as gene-level. Principal component analysis (PCA) was used to identify outliers. Differential expression analysis comparing HFD and CF at the gene and transcript level of summarization were then carried out using open source R package Ballgown. Analysis were adjusted for multiple testing by reporting the FDR q values for each feature and *q* < 5% declared as genome-wide significant. R statistical software environment using the GAGE Bioconductor packages were used to carry out the analysis on pre-defined ontology (GO) gene sets by conducting two sample *t*-tests on the log based fold changes of target gene set and control sets. Both up- and down-regulated gene sets are reported on three GO sub-categories: Biological process (BP), Cellular component (CC), and molecular Function (MF). FDR q-values were estimated to correct the *p*-values for the multiple testing issue.

### Statistics

All results are analyzed using Graph Pad Prism version 7.0 and expressed as means with error bars reflecting SD or SEM as indicated. *n* represents the number of animals per experiment. Differences between two groups were assessed using unpaired two-tailed Student's *t-*tests. A one-way or two-way ANOVAs with Tukey's or Sidak's multiple comparison *post hoc* tests were applied for analysis of data from more than two groups.

## Results

### Obesity Accelerates Proliferation of DC-Restricted Progenitors

**Figure 1:**
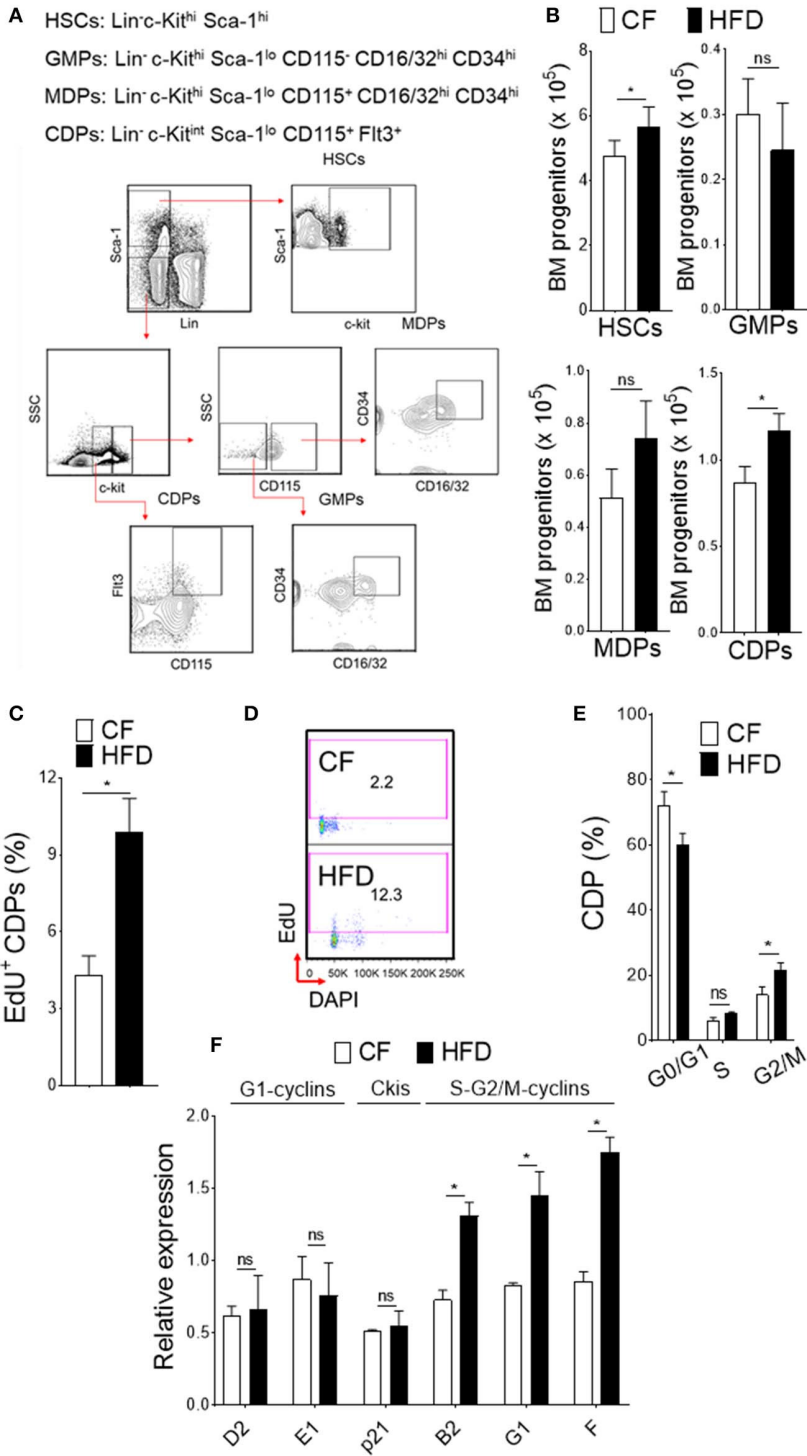
DC restricted precursor maintain a highly proliferative state in obesity. **(A)** Phenotypic definition and gating strategy of BM progenitors. **(B)** Enumeration of progenitors in diet-induced obese mice (HFD) compared with chow fed (CF) control mice. *n* = 10/group. **(C)** Mean percentages of EdU (5-ethynyl-2′-deoxyuridine) showing proliferation of BM CDPs from EdU-treated CF and HFD mice. *n* = 5/group. Two independent experiments. Results are expressed as mean ± s.e.m. **(D)** Representative FACS plot and **(E)** mean percentages of cell-cycle profile showing CDPs from CF and HFD mice. *n* = 5 mice/group. **(F)** qRT-PCR analyses for G1-cyclins (D2, E1), Ckis (p21), and S-G2/M-cyclins (B2, G1, and F) gene expression in BM CDPs isolated from CF and HFD mice. Two independent experiments. Results are expressed as fold change ± s.e.m normalized to β-actin expression. *n* = 4/group. Student's *t*-test *P < 0.05.

Obesity-induced burdens arise from low level inflammation and immune dysfunction (34). One key factor to the development of obesity in humans is a high-fat diet. To fully recapitulate its phenotype, we conducted our studies in WT mice fed with high fat diet (HFD). Mice in this model were fed up to 16 weeks with 60 kcal% fat and as such analyzed for experiments. At this time point, we observed increase cellularity in BM and analyzed the progenitor population from HFD and normal chow-fed (CF) mice (10 kcal% fat) (Figures 1A,B). In HFD-fed mice, we noticed increase in absolute number of HSCs (Lin^−^ c-Kit^hi^ Sca-1^hi^) and DC-restricted progenitors (CDPs; Lin^−^ c-Kit^int^ Sca-1^lo^ CD115^+^ Flt3^+^) in BM compartment. Conversely, the numbers of GMPs (granulocytes monocytes progenitors; Lin^−^ c-Kit^hi^ Sca-1^lo^ CD115^−^ CD16/32^hi^ CD34^hi^) or MDPs (monocyte dendritic cell progenitors; Lin^−^ c-Kit^hi^ Sca-1^lo^ CD115^+^ CD16/32^hi^ CD34^hi^) remained unchanged, suggesting an impaired differentiation process of the progenitor cell niche by HFD. We then used comprehensive proliferation analysis of CDPs with intraperitoneal EdU injection in mice. In obesity, we found increase accumulation of EdU (Figure 1C) and higher percentage of EdU/DAPI-double stained CDPs (Figure 1D), suggesting increased proliferation capacity of DC-restricted progenitors. These changes in proliferation capacity of DC-restricted progenitors were reflected in cell-cycle phase analysis (Figure 1E). Obesity was associated with decrease quiescence and reduction in the frequency of G0/G1 phase, correlating with an increase in the frequency of cycling G2/M phase of CDPs. To gain further insight on the effect of obesity on cell cycle regulation in DC-restricted progenitors, we assessed the expression levels of selective components and regulators of cell cycle machinery by quantitative RT-PCR in flow sorted CDPs (Figure 1F). Interestingly, we found specific up-regulation of S-G2/M cyclins from CDPs in HFD-fed mice, indicating that obesity enable activation of genes required for S phase entry and progress through mitosis. Altogether, our results indicate that obesity substantially impaired steady-state hematopoiesis in the BM compartment and lead to an overall increased proliferation of DC-restricted progenitors.

### Obesity Is Associated With Activation of p38 MAPK in DC-Restricted Progenitors

**Figure 2:**
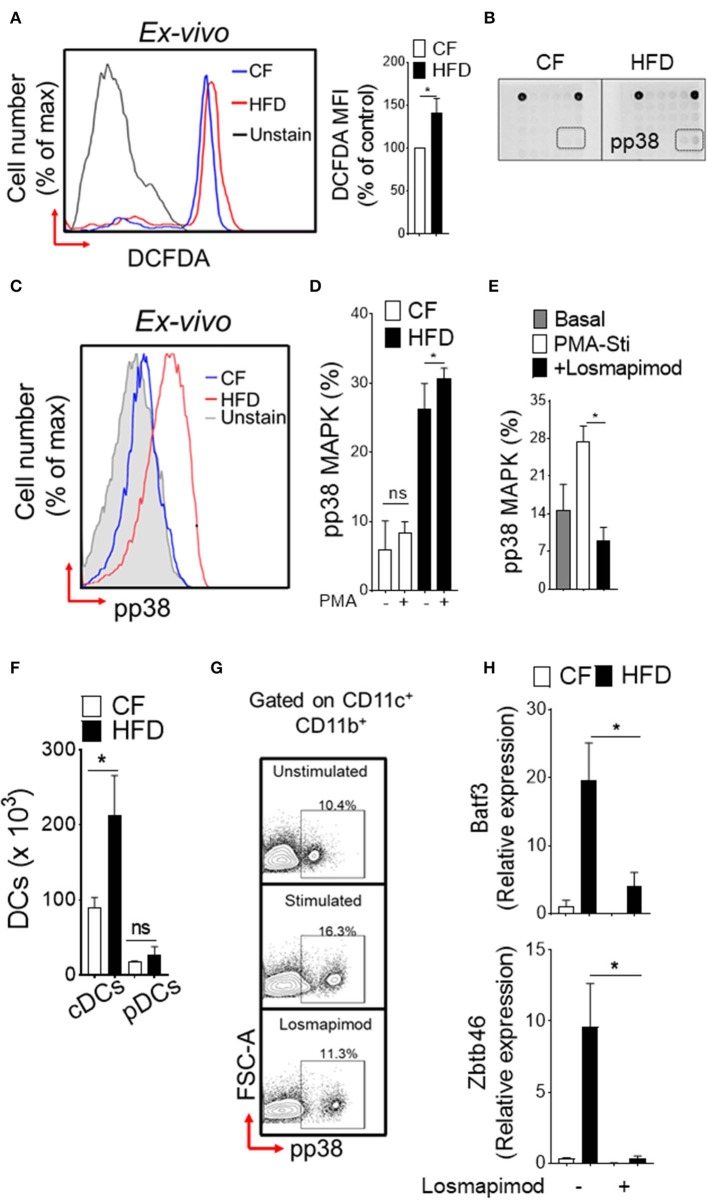
Induction of p38 MAPK in DC restricted precursor is associated with obesity. **(A)** Representative FACS plots (left) and mean fluorescence intensity (MFI) ± s.e.m (right) showing endogenous reactive oxygen species (ROS) level in DC-restricted CDPs from CF and HFD as measured by redox sensitive probe CM-H2DCFDA. **(B)** The phosphorylation status of 18 different signaling proteins in purified CDPs by array hybridization. **(C)** Representative FACS plots and **(D)** bar graph of mean percentages showing basal and PMA-stimulated *ex vivo* levels of phosphorylated p38 in CDPs from CF and HFD mice. *n* = 5/group. Two independent experiments. **(E)** Bar graph of mean percentages of pp38 MAPK in CDPs isolated from HFD and cultured in presence of losmapimod (20 μM). *n* = 4 biological replicate. Results are expressed as mean ± s.e.m. one-way ANOVA with Sidak's multiple comparison test *p < 0.05. **(F)** Numbers of splenic DCs subset in CF and HFD mice and **(G)** representative FACS plots of cultured DCs from HFD mice. Isolated DCs were treated with potent p38α/β MAPK inhibitor losmapimod (20 μM) and gated on CD11c^+^ CD11b^+^ cells. **(H)** qRT-PCR analyses of lineage-specific transcription factors in DCs isolated from CF and HFD mice. Data represents fold change ± s.e.m normalized to GAPDH expression. Three sets of independent experiment. One-way ANOVA with Sidak's multiple comparison test *P < 0.05.

To explore the functional consequences of obesity-associated changes, we next assessed the oxidative stress in DC-restricted progenitors and downstream signaling pathways. We found a significantly increased level of reactive oxygen species (ROS; Figure 2A) in DC precursors isolated from HFD mice. Screening of intracellular pathways in purified CDPs by array hybridization, we found that p38 MAPK were phosphorylated with HFD (Figure 2B). As expected, HFD led to a basal level increase in phosphorylation of p38 MAPK as compared to CF in DC precursors (Figure 2C). PMA-stimulation did not affect further p38 MAPK phosphorylation in DC- progenitors (Figure 2D), suggesting that oxidative stress induces persistent activation of these pathway in obesity. As expected, the treatment with p38α/β MAPK-specific inhibitor losmapimod led to a normalization of p38 MAPK phosphorylation in DC precursors from HFD (Figure 2E). In parallel, we observed increased number of precursor-derived DCs in peripheral tissues (Figure 2F), suggesting that HFD drives the differentiation process. Notably, activation of p38 MAPK (Figure 2G) and lineage-specific expression of transcription factors (Figure 2H) were reversed by treatment with losmapimod, indicating that a persistent p38 MAPK signaling during DC differentiation process were modulated in obesity. Therefore, these results indicate that a tight control of p38 activation is critical to DC-restricted progenitor function, particularly during stress, and show that p38 MAPK signaling is one of the key determinants in CDP function in obesity.

### Obesity Promotes an Aberrant Airway Inflammatory Response to Inhaled HDM Challenge

**Figure 3:**
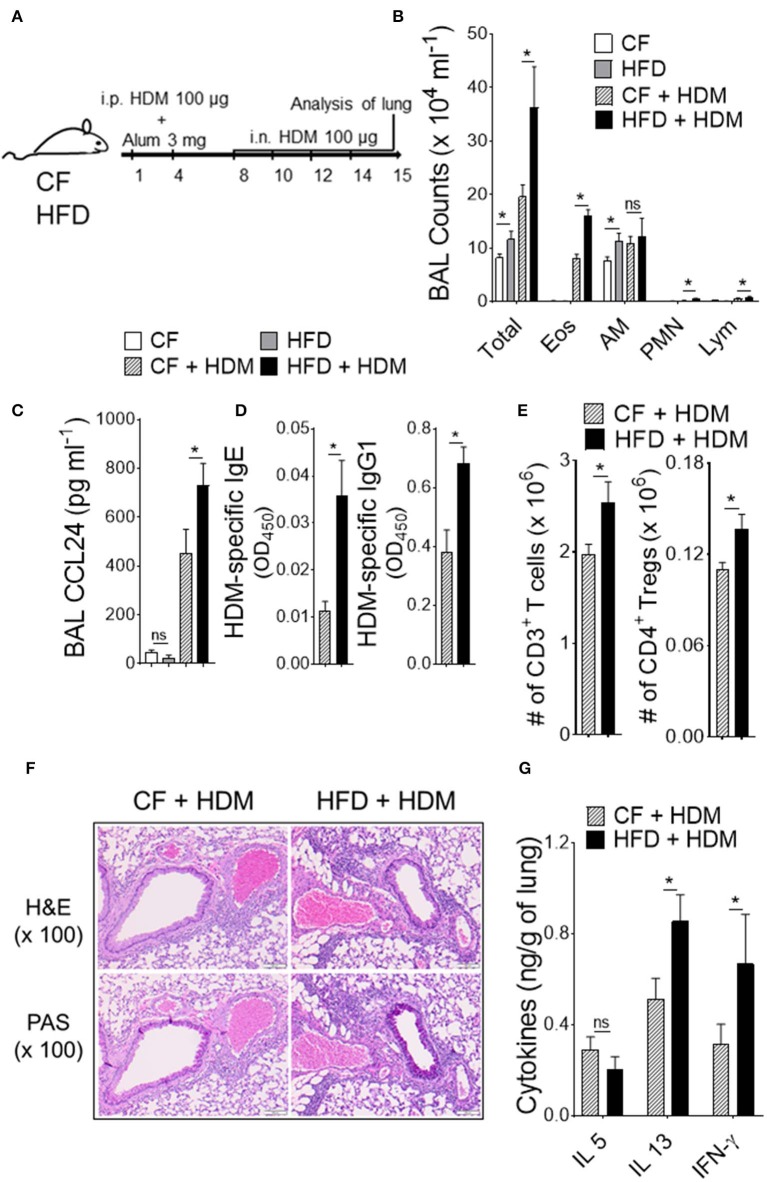
Obesity increases the severity of airway inflammation. **(A)** Timeline for sensitization and challenge. CF or HFD mice were immunized with HDM, as indicated. *i.n*., intranasal; *i.p*., intraperitoneal. **(B)** Differential cell counts and **(C)** CCL24 in BAL. **(D)** Plasma levels of HDM-specific IgE and IgG1 assessed by ELISA. **(E)** Enumeration of total T cells and Tregs from lung draining mediastinal lymph nodes. **(F)** Hematoxylin and eosin (H & E) and periodic acid-schiff (PAS)-stained lung sections (Scale bars 100 μm for 100x images). **(G)** Lung cytokine levels were measured by means of ELISA. Data are representative of two independent sets of experiment and expressed as mean ± s.e.m. *n* = 8–10/group. One-way ANOVA with Sidak's multiple comparison test *P < 0.05. *Eos*, Eosinophils; AM, Alveolar macrophage; PMN, Neutrophils; Lym, Lymphocytes; Tregs, T regulatory cells.

Obesity is a proven risk factor for allergic airway diseases, such as asthma. Asthma severity appears to be increased in the obese and alters the immune response (19). To explore the functional consequences of the specific changes in DC-restricted progenitors by HFD, we investigated lung changes using experimental murine model of allergic airway inflammation (Figure 3). We sensitized and challenged CF or HFD-fed mice with or without innocuous aeroallergen HDM (Figure 3A). Analysis of the bronchoalveolar lavage (BAL) showed a dramatic increase in total number of inflammatory cells, which represented increases in eosinophils, neutrophils and lymphocytes in HDM-challenged mice with HFD (Figure 3B). BAL levels of CCL24 (eotaxin-2), a surrogate marker of eosinophilia, were significantly increased in allergic HFD lungs (Figure 3C). Analysis of peripheral blood showed dramatic increase in HDM-specific IgE and IgG1 levels in HFD-fed allergic mice (Figure 3D). The number of CD3^+^ T cells in lung draining lymph nodes, specifically the regulatory T cells (Tregs) were strikingly increased with HDM challenge and HFD (Figure 3E). Lung histopathology showed an increase in the peribronchial inflammatory cell infiltrates scattered around large conducting airways and mucous cell metaplasia in allergen-challenged obese mice (Figure 3F). Analysis of lung cytokines showed increased IL13 and IFN-γ but not IL5 levels (Figure 3G). Altogether, these results show that obesity exacerbates HDM-induced allergic airway inflammation and triggers lung–intrinsic Th1/Th2 immune responses.

**Figure 4:**
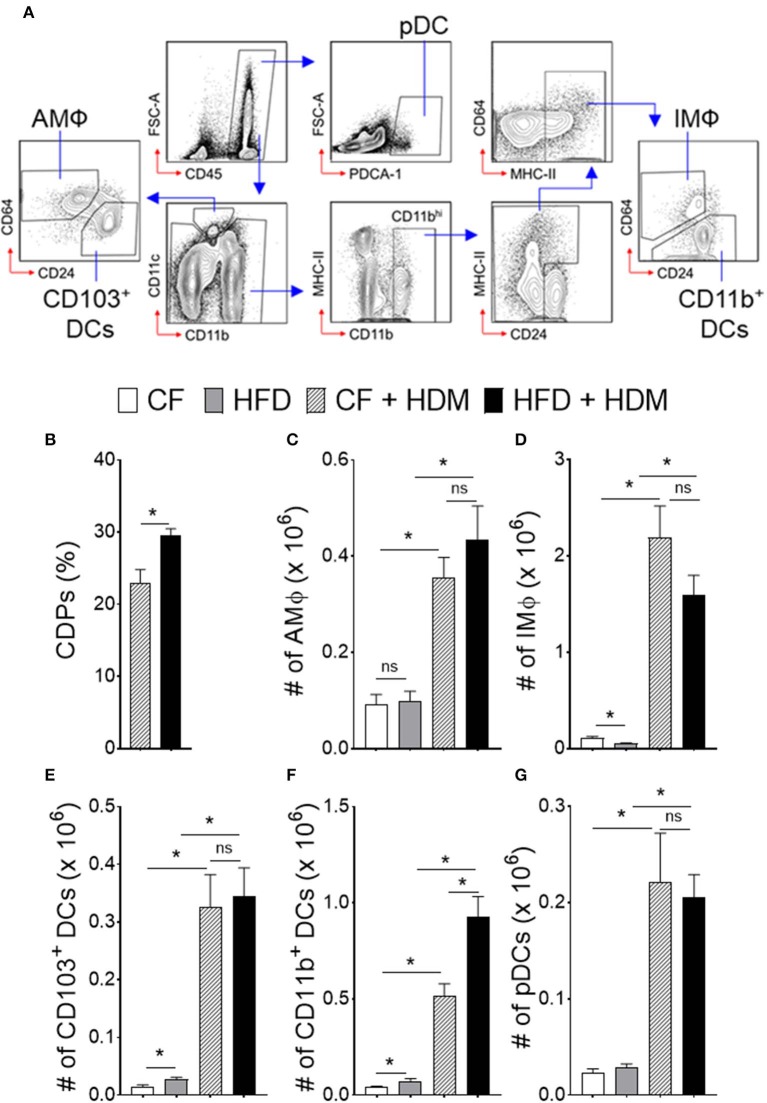
Obesity alters myeloid cell accumulation in airway inflammation. Naïve HFD and CF mice were sensitized, as mentioned in Figure 3. One day after the last HDM sensitization, lungs were analyzed for different subsets of antigen presenting myeloid cells. **(A)** FACS plot show the gating strategy. Blue arrows show the gating hierarchy. **(B)** Bar graph showing mean percentages of CDPs in the BM and **(C)** number of alveolar macrophages, **(D)** interstitial macrophages, conventional **(E)** CD103^+^ DCs, and **(F)** CD11b^+^ DCs and **(G)** plasmacytoid DCs in the lung. Data are representative of two independent sets of experiment and expressed as mean ± s.e.m. *n* = 8–10/group. One-way ANOVA with Sidak's multiple comparison test. *P < 0.05. AMΦ, Alveolar Macrophage; IMΦ, Interstitial Macrophage; pDCs, Plasmacytoid DCs.

We next investigated DC-restricted progenitors in the BM compartment and recruitment of myeloid cells in the lung that could contribute to the abnormal function in obesity and airway inflammation (Figure 4). Increased number of CDPs in the BM was found in HFD-fed but not in CF mice with allergen challenge (Figure 4B). In parallel, we observed enhanced recruitment of myeloid cells, such as alveolar macrophage (AMΦ), interstitial macrophage (IMΦ), CD103^+^ DCs, CD11b^+^ DCs, and plasmacytoid DCs (pDCs) from both CF and HFD fed allergic mice as compared to non-allergic control, suggesting an enhanced demand of myeloid cells in airway inflammation (Figures 4C–G). Lung CD11b^+^ DCs, which were further distinguished from CD64^hi^ expressing IMΦ were significantly increased with HFD but not in CF allergic mice. However, this differences appear modest in lung AMΦ, CD103^+^ and pDCs recruitment. As such, these results further show that a significant number of myeloid CD11b^+^ DCs that induce allergic airway inflammation in response to inhaled HDM are driven by HFD and are constantly replenished by the BM compartments. Collectively, these results demonstrate that obesity drives airway inflammation that includes enhanced recruitment of DCs in the lungs.

### Obesity Is Associated With Up-Regulation of Adam17 Expression in DC-Restricted Progenitors

**Figure 5:**
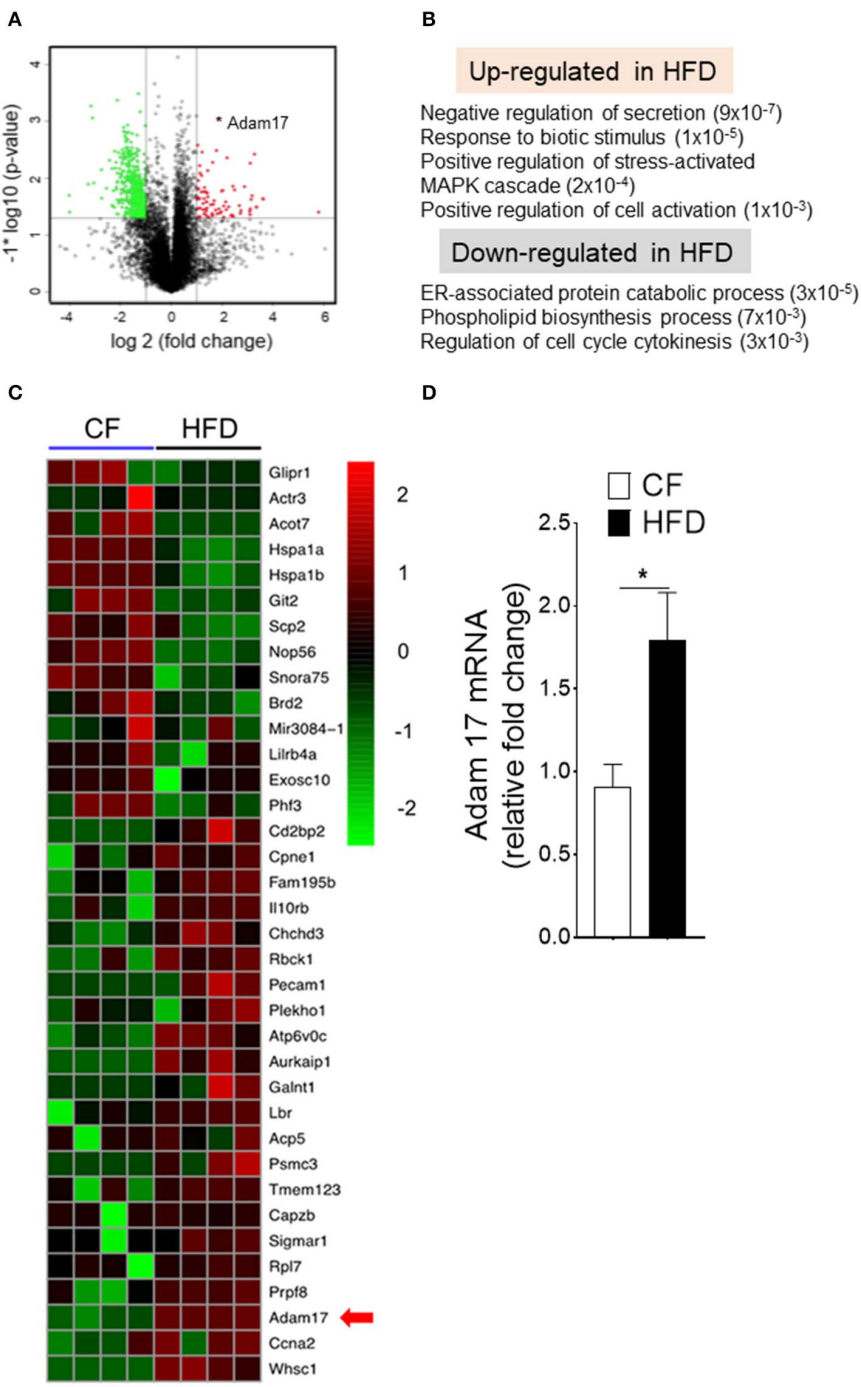
Obesity regulates Adam17 expression in DC-restricted progenitors. **(A)** Volcano plot of log2 fold changes vs. one-way ANOVA *p*-value (–log10 scale) between expressions of mRNA isolated from BM CDPs of CF and HFD mice. Each dot represents one transcript. The transcript of Adam17 is indicated with an asterisk. **(B)** GSEA for dysregulated genes in HFD CDPs. Numbers in parenthesis indicate adjusted *p*-values. **(C)** Heat map of differentially expressed genes based on transcript-level RNA-seq results. Top 70% of genes with a *p*-value < 0.05 and the corresponding log2 fold change >2 or <-2 are shown. **(D)** qRT-PCR analyses for Adam17 gene expression in CDPs isolated from CF and HFD mice. Results are expressed as fold change ± s.e.m normalized to β-actin expression. *n* = 4/group. Student's *t*-test *P < 0.05.

We next investigated the molecular mechanisms that could influence to the enhanced proliferation of DC-restricted progenitors in obesity. We performed genomewide gene expression analysis on CDPs isolated from CF and HFD mice (Figure 5). Gene set enrichment analyses (GSEAs) showed a specific up-regulation in DC-restricted progenitors of genes associated with signaling pathway (regulation of MAPK cascade and cell activation), a set of function potentially related to their increased proliferation and differentiation (Figure 5B). In parallel, the down-regulation of genes linked with several stress response (ER-associated protein catabolism process, phospholipid biosynthesis process, and cell-cycle regulation), suggesting DC-restricted progenitors were directly modulated by environmental stresses associated with obesity. We then focused our analysis on progenitor cells-relevant genes whose expression was affected by HFD. Among the differentially expressed genes in HFD (Figure 5C), we noted transmembrane protease Adam17, a disintegrin, and metallopeptidase domain 17 (Adam17) known to regulate many substrates in cell-cell interaction and cell proliferation. We confirmed by quantitative RT-PCR (qRT-PCR) analyses the higher expression of Adam17 in DC-restricted CDPs isolated from the HFD (Figure 5D). As such, these results further show that HFD is a key contributor to the dysregulation of Adam17 expression and is an obesity-response gene in DC-restricted progenitors.

### Adam17 Up-Regulation Mediates p38 MAPK Activation and Expansion of DC-Restricted Progenitors in Obesity

**Figure 6:**
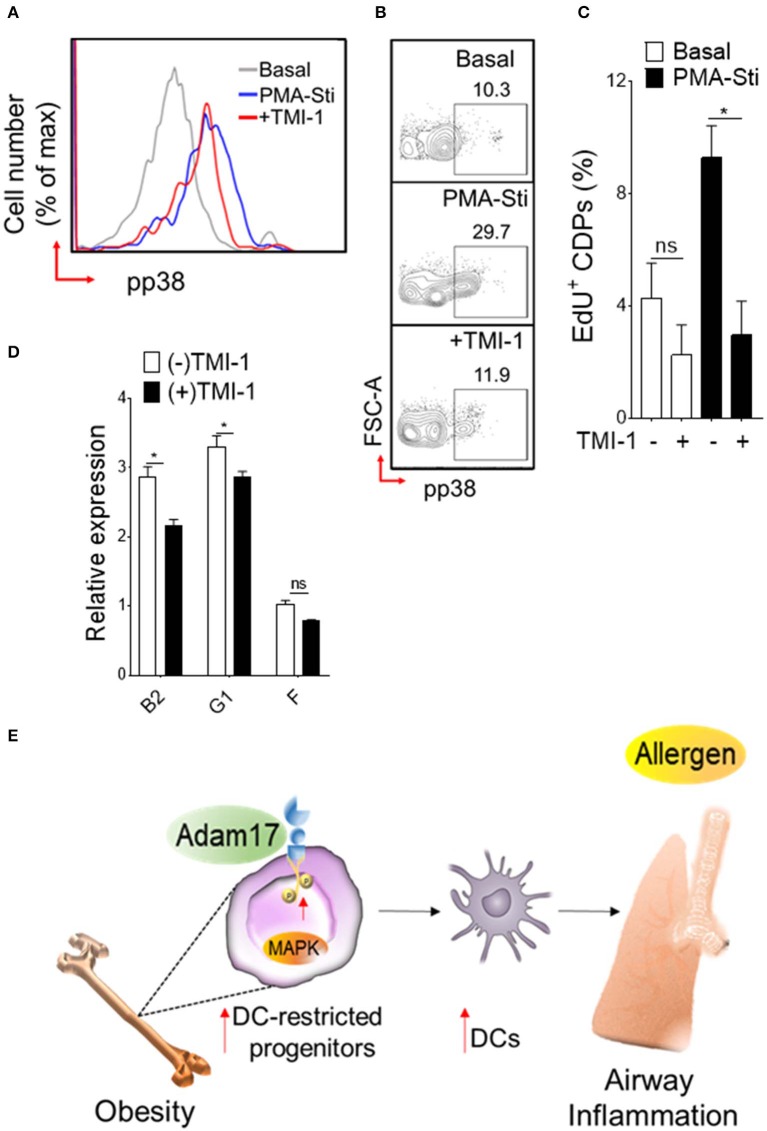
Adam 17 up-regulation in obesity affects DC-restricted progenitors. **(A)** Histogram and **(B)** representative FACS plots showing basal and PMA-stimulated levels of phosphorylated p38 and **(C)** mean percentages of EdU (5-ethynyl-2′-deoxyuridine) showing proliferation of CDPs treated with selective Adam 17 inhibitor. BM hematopoietic stem cells were isolated from HFD mice and cultured overnight in presence of Adam 17 inhibitor TMI 1 (50 μM). Phosphorylation and proliferation analyses showing on gated LSK^+^ CD115^+^ Flt3^+^ cells. **(D)** qRT-PCR analyses for S-G2/M-cyclins (B2, G1, and F) gene expression in cultured cells isolated from HFD mice. Data expressed as mean ± s.e.m. *n* = 4. Student's *t*-test *P < 0.05. **(E)** Schematic representation of proposed Adam 17-p38 MAPK signaling in DC-restricted progenitors in obesity-associated airway inflammation.

Diet-induced obesity generates both quantitative increases in CDPs and cell surface expression of Adam17. To determine whether Adam17 up-regulation by HFD in DC-restricted progenitors directly affects the downstream p38 MAPK pathway and proliferation, we employed pharmacological inhibition of Adam17 and assessed p38 phosphorylation and proliferation assays (Figure 6). Normalization of p38 MAPK phosphorylation was found when progenitors isolated from HFD mice were treated *in vitro* with Adam17 inhibitor (Figures 6A,B), indicating that a direct effect of Adam17 activation on p38 MAPK phosphorylation in DC-restricted progenitors. A similar effect was found with Adam17 inhibitor (Figure 6C), suggesting that proliferation of DC precursors could be regulated by Adam 17 activation in obesity. Finally, we found that expression of S-G2/M cyclins in DC-restricted progenitors after Adam17 inhibitor treatment were significantly decreased (Figure 6D). Altogether, these results indicate that a tight control of Adam17 expression is critical to dysregulation of p38 MAPK signaling particularly in obesity, and show that Adam17 is one of the key determinants of DC-restricted progenitor expansion.

## Discussion

Using dietary mouse model, our results demonstrate that obesity has a regulatory effect on lineage-specific dendritic-cell progenitors in the BM compartment. Although DC-restricted CDP population display a limited perturbation at steady-state, they show an exacerbated proliferative response and expansion in obesity along with quantitative increase of lung myeloid cells in airway inflammation. Mechanistically, we found that this aberrant CDP activity in BM is linked to the acquisition of specific molecular features, particularly activation of p38 mitogen activated protein kinase (MAPK) signaling and up-regulation of a disintegrin and metallopeptidase domain 17 (Adam17) expression. We show that these molecular changes of DC-restricted progenitors in obesity-associated low-grade inflammation respond unequivocally to allergen—induced asthma and impairs the distribution of lung myeloid cells. Finally, we demonstrate that Adam17 is one of the key regulators of p38 MAPK activation and is required for the proliferation of DC-restricted progenitors in obesity. In sum, we establish a mechanistic link between enhanced demand of DCs and exacerbation of allergic asthma in the setting of obesity (Figure 6E).

Expansion of HSCs and oxidative stress response are hallmark of obesity-associated changes (14, 34). In parallel our results demonstrate quantitative expansion of lineage-specific niche cells in the BM pool (38). We found increased levels of intracellular ROS generation in DC-precursors promoting a rapid proliferation through induction of the p38 MAPK. This suggest a progressive acquisition of oxidative stress in obesity that leads to specific adaptive changes in DC-restricted progenitors (39). In this study, we show that oxidative stress and p38 MAPK activation in obesity positively correlates with the proliferation and expansion of DC-restricted progenitors. We found a small fraction of actively dividing CDPs that correlates with induction of mitotic S-G2/M cyclins in obesity. This is consistent with prior reports demonstrating that HFD profoundly effects the BM microenvironment and dynamics of stem and progenitor cell expansion and differentiation (15, 40). Importantly, the MAPK family of serine/threonine kinases are an integral part of progenitor cell signaling in response to stressors and able to fine-tune the balance between expansion, survival and differentiation process (41). As such, mouse deficient in cell-cycle checkpoint regulator Atm undergo constitutive activation of p38 MAPK signaling and higher ROS generation resulted in HSC exhaustion and sustain hematopoiesis (39, 42). Notably, the HSC phenotype in obesity is driven by specific changes in adipose tissues in the BM compartment and remain to be fully established. Further, p38 MAPK positively regulates proliferation of HSC-derived progenitors including erythroid progenitors (43) myeloid progenitors (44) and endothelial progenitors in obesity (45). As others, we found sustained p38 MAPK activation in DC precursors and DCs isolated from HFD mice. We show that obesity associated changes primes the DC precursors with persistent p38 activation and expansion that could be reversed by pharmacological inhibition of p38 activity. This suggest that alterations of DC supply chain contribute to the chronic low-grade inflammation associated with obesity (46, 47).

In this study, we show that p38 activation associated with obesity directly impacts on Adam17 expression in DC-restricted progenitors. Adam17 expression is tightly controlled in obesity as Adam17-knockout mice on HFD shows protection from insulin resistance, diabetes, non-alcoholic fatty liver disease and tumorigenesis (48, 49). Global deletion of Adam17 gene in mice shows developmental defects due to impaired EGFR and TNF-α receptor signaling and are characterized by lean, hypermetabolic phenotype (50), suggesting a pivotal role of Adam17 in energy homeostasis. As such, Adam17 act as a key regulator to fine-tune the properties of DC-restricted progenitors. In obesity, we show that Adam17 plays a regulatory role contributing to reinforce p38 MAPK activation and proliferation of DC-restricted progenitors. Importantly, modulation of Adam17 expression through potent Adam 17 inhibitor is effective in restoring p38-mediated proliferation and mitotic S-G2/M cyclin expression in CDPs. However, it remains uncertain whether S-G2/M cyclin expression directly linked to Adam 17 activity and actually contributes to CDP properties (51). Collectively, these results demonstrate that Adam17-p38 MAPK axis is tightly coupled to proliferation in DC-restricted progenitors in obesity.

Diet-induced obesity can modify the immunological environment in the lung and influence the severity of airway inflammation (8, 9, 18, 52). There are substantial precedents to suggest many complementary and synergistic pathways of obesity-induced or obesity exacerbated asthma (6, 19), however, its impact on dendropoiesis remains poorly understood. Finally, our work establishes that allergic airway inflammation in obesity leads to alterations in the BM compartment and potentiate generation of proinflammatory lung DCs. In consistent with others, we found that HFD exacerbates airway inflammation, as indicated by an increase in BAL cellularity, plasma IgE, and IgG1 levels, and Th2/Th1 immune response (19, 53, 54). We show lung CD11b^+^ conventional DCs, which are specialized for antigen presentation and to generate T cell-mediated immune responses in allergic asthma (55–57), notably increased by HDM sensitization. Thus, the status of airway inflammation during established obesity dramatically differs from non-obese lung environment and is supported by quantitative and qualitative alterations in myeloid populations. Molecularly, obesity-associated changes in DC precursors is supported by an accumulation of lung DCs in HDM-induced airway inflammation.

In summary, these data demonstrate a mechanistic link between DC precursors and exacerbation of allergic asthma in the setting of obesity. We show that diet-induced obesity impairs proliferation of DC-restricted progenitors via Adam17-p38 MAPK-dependent pathway. Furthermore, the obesity-associated changes in DC precursors from BM and DCs elicit an impaired immune response in allergic asthma. Thus, given the central role of DCs in allergic asthma and their rapid turnover in airways, interrupting the DCs supply chain may attenuate asthma severity in obesity.

## Data Availability Statement

The data generated for this study have been deposited at the Gene Expression Omnibus (GEO) under accession code GSE144335 (https://www.ncbi.nlm.nih.gov/geo/query/acc.cgi?acc=GSE144335).

## Ethics Statement

The animal study was reviewed and approved by Auburn University Animal Care and Use Committee.

## Author Contributions

AJ, AM, and SL designed the experiments. AM together with AJ, ASa, SM, NS, ASu, PD, and MS performed the experiments. AM, AJ, ASa, ASu, JM, and MS analyzed the data. AM and AJ wrote the manuscript.

### Conflict of Interest

The authors declare that the research was conducted in the absence of any commercial or financial relationships that could be construed as a potential conflict of interest.
